# Supplementary material for: Ammonium-Generating Microbial Consortia in Paddy Soil Revealed by DNA-Stable Isotope Probing and Metatranscriptomics
Source: Microorganisms. 2025 Jun 21;13(7):1448. doi: 10.3390/microorganisms13071448 (PMC12298685; doi:10.3390/microorganisms13071448)
Supplement: Supplementary file 1 [file microorganisms-13-01448-s001.zip › microorganisms-3643459-supplementary.pdf]

Supplementary Text

“Ammonium Generating Microbial Consortia in Paddy Soil Revealed by the Combination of DNA-Stable Isotope Probing and Metatranscriptomics” by Wang et al.

S1. Detection of N_2O content

For N_2O detection, 1 mL of headspace gas in the bacteria cultural bottles was withdrawn by a sterile syringe and then mixed with 19 mL air gas in a 20-mL vacuum vial. The mixed gas in the vials was automatically measured by a gas chromatography with an electron capture detector (GC-ECD, GC-2014, Shimadzu, Kyoto, Japan).

S2. Detection of soil ammonium content

Ammonium content in soil was determined using the phenol-hypochlorite method [27]. Briefly, 4 g of soil sample was mixed with 10 mL of 2 M KCl solution and shaken at 25°C for 30 min to extract ammonium ions. The suspension was then filtered through a 3 μm filter paper, and the filtrate was collected for analysis. A standard curve was established using ammonium solutions with concentration of 0, 0.048, 0.096, 0.192, 0.384, and 0.768 mg/L. For colorimetric detection, 2.5 mL of each sample or standard solution was mixed with 1 mL of 0.1 g/mL phenol-hypochlorite reagent, 1 mL of 0.05 g/mL sodium nitroprusside reagent, and 2 mL of oxidizing reagent (0.05 g/mL sodium hypochlorite in alkaline citrate solution). After incubation at room temperature for 20–30 min, the absorbance was measured at 640 nm using a UV-VIS spectrophotometer (UV-1900, Shimadzu, Japan), and ammonium concentrations were calculated based on the standard curve.

Supplementary figures

"Elucidation of Ammonium Generating Microbial Consortia via Reductive Nitrogen Transformation in Paddy Soil Revealed by the Combination of DNA-Stable Isotope Probing and Metatranscriptomics" by Wang et al.

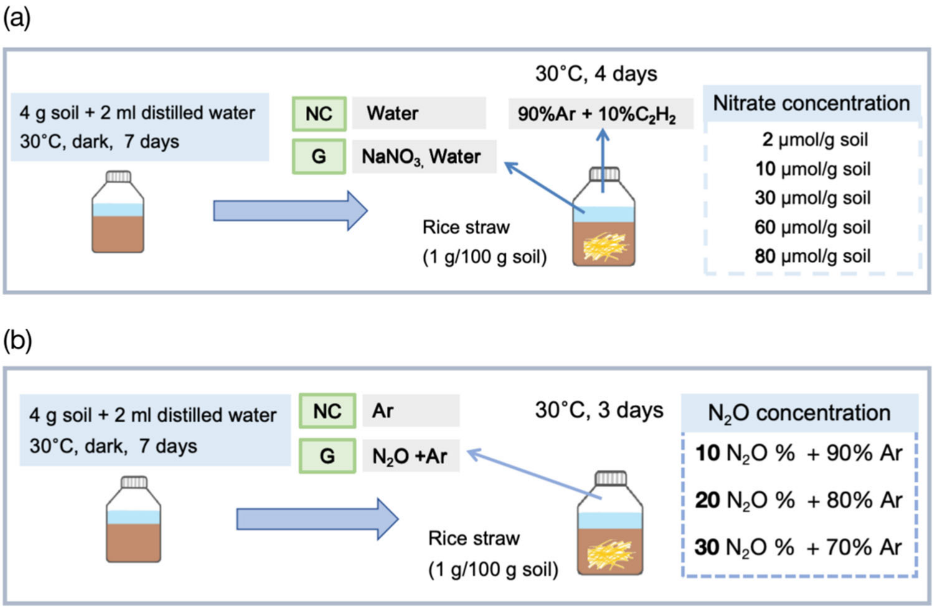

Figure S1. Experimental design (a) nitrate and (b) N_2O concentration gradient treatments. The amount of nitrate added was from 2 μmol / g soil to 80 μmol / g soil, and the gas phase of the vials was filled with 90% Ar + 10% C_2H_2. The N_2O concentration was from 10% to 30%, and the negative control received only straw and the gas phase was Ar. The straw concentration was 1.0 g/g mixed soil. Three replicates were set up for each treatment group.

(a)

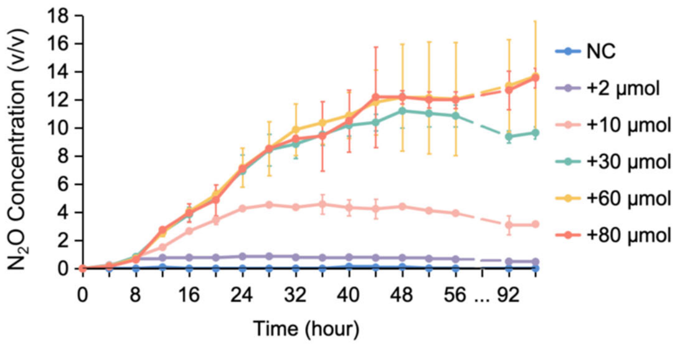

(b)

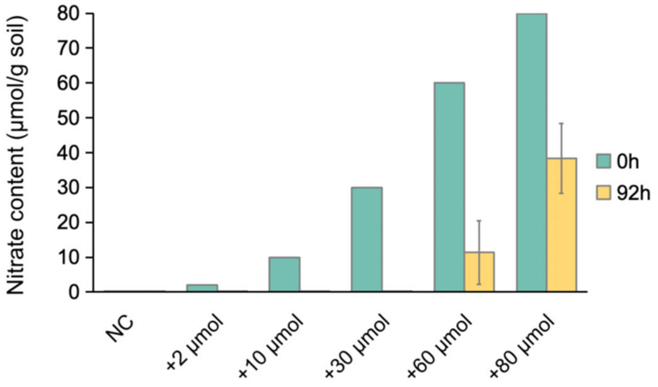

Figure S2. Comparison the change of nitrous oxide accumulation in microcosms under different concentrations of nitrate treatment with time. Data are shown as the average of triplicates. The x-axis represents the incubation time; the y-axis indicates the proportion of N_2O within the total gas composition in the incubation bottles (a). Comparison of nitrate content remaining in microcosms under different concentrations of nitrate treatment after 0 h and 92 h of incubation. Data are shown as the average of triplicates. The x-axis represents the treatment groups with different concentrations of added nitrate (from 2 μmol $NaNO_3$ / g soil to 80 μmol $NaNO_3$ / g soil), NC: negative control; the y-axis represents the nitrate content in the incubated soil samples (b).

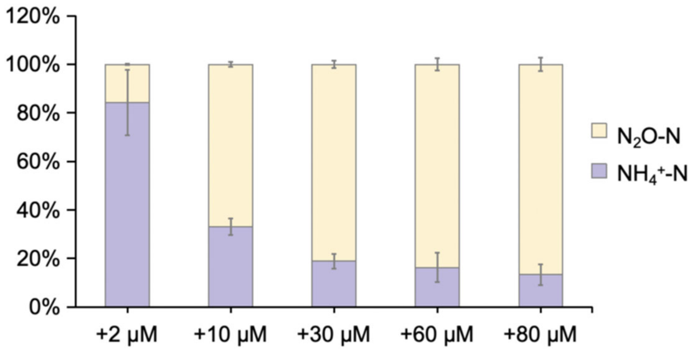

Figure S3. Comparison of the amount of nitrogen in added nitrate converted to nitrogen in ammonium (NH_4^+) and nitrous oxide (N_2O), respectively, in the groups treated with different concentrations of nitrate after 92 hours of incubation. The x-axis represents the treatment groups with varying concentrations of added nitrate (from 2 μmol $NaNO_3$ / g soil to 80 μmol $NaNO_3$ / g soil); the y-axis represents the proportion (%) of nitrogen from nitrate that was converted into nitrous oxide (N_2O) and ammonium (NH_4^+). Data are shown as the average of triplicates.

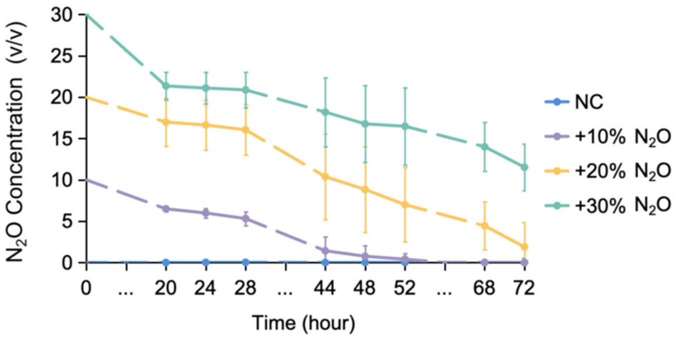

Figure S4. Comparison the change of nitrous oxide concentration in microcosms under different concentrations of nitrate treatment with time. Data are shown as the average of triplicates.

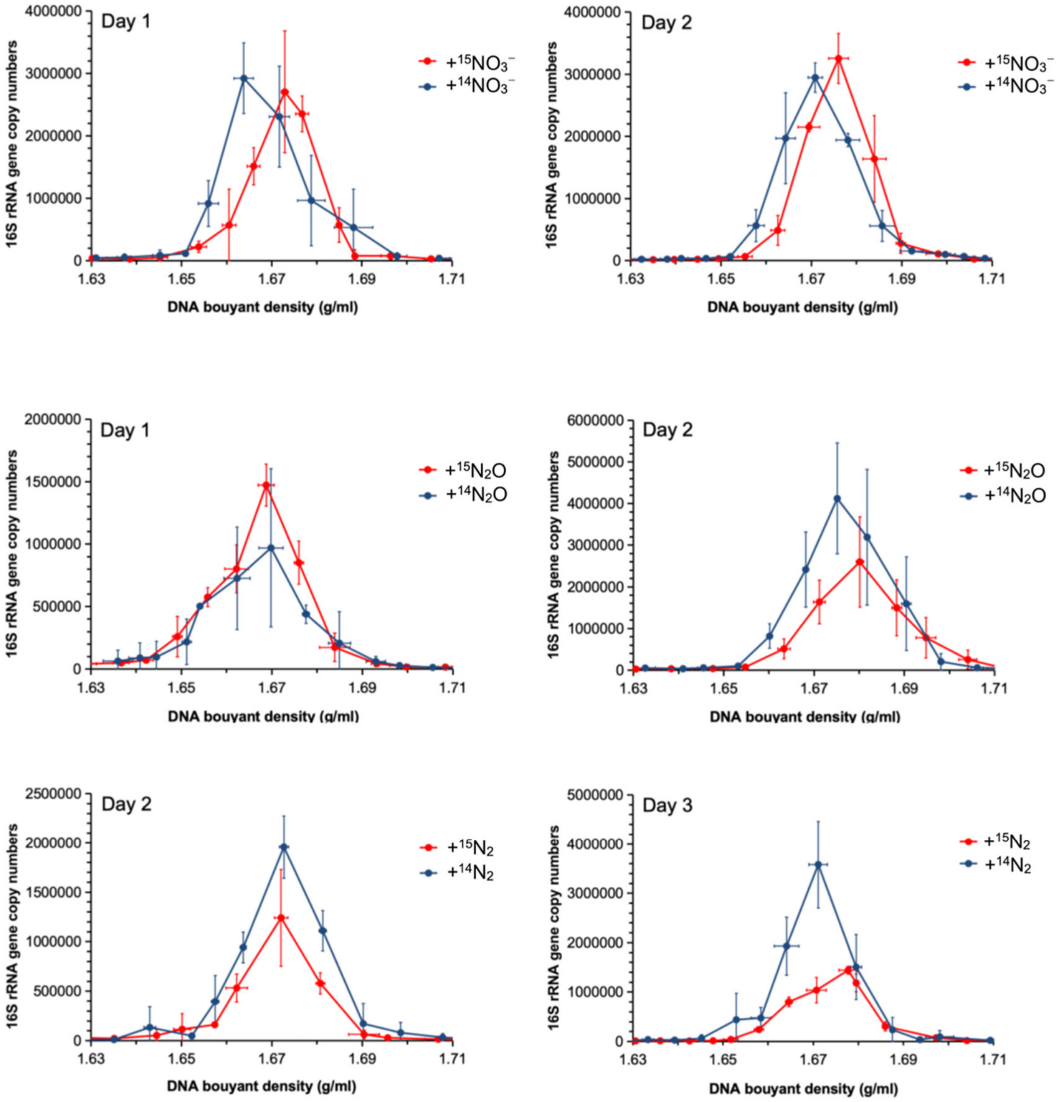

Figure S5. Absolute copy numbers of the 16S rRNA gene quantified by qPCR across CsCl gradient fractions from the +$^{15}NO_3^-$, +$^{15}N_2O$, and +$^{15}N_2$ treatments after 1-3 days of incubation. The x-axis indicates buoyant density (g/ml), and the y-axis shows the absolute copy number of the 16S rRNA gene determined by qPCR. Data are presented as the average of triplicate measurements. Vertical error bars represent the standard error of relative abundance, while horizontal error bars represent the standard error of buoyant density for corresponding fractions.

Supplementary tables

"Elucidation of Ammonium Generating Microbial Consortia via Reductive Nitrogen Transformation in Paddy Soil Revealed by the Combination of DNA-Stable Isotope Probing and Metatranscriptomics" by Wang et al.

Table S1. Physicochemical characteristics of the soil

	**Soil type**	**Clay (100%)**	**Silt (100%)**	**Sand (100%)**	**Total C (g kg-1)**	**Total N (g kg-1)**	**NO_3^- (μg/g dry soil)**	**NH_4^+ (μg/g dry soil)**
Nagaoka	Gray Lowland soil	35.8[b]	29.0[b]	35.2[b]	11.0[b]	1.09[b]	3.02[a]	28.42[a]

a: from this study; b: from [25].

Table S2. PCR reaction for 16S rRNA amplicon sequencing

(a) PCR reaction system

**Reagent**	**Amount**
10× Ex buffer	10 μL
dNTPs (each 2.5 mM)	1.6 μL
Forward primer (10 μM)	1.0 μL
Reverse primer (10 μM)	1.0 μL
Ex Taq (5 units/μl)	0.2 μL
Milli-Q water	13.2 μL
Template DNA	1.0 μL
Total	20 μL

(b) PCR reaction system

**Temperature**	**Time**	**Cycles**
94.0 °C	2 min	1
94.0 °C	30 sec	25-35
55.0 °C	30 sec	
72.0 °C	30 sec	
72.0 °C	5 min	1

Table S3. Sequence identity thresholds for 16S rRNA gene used to assign taxonomic ranks

Taxonomic Rank	Sequence Identity (%)
Species	≥ 98.7
Genus	≥ 94.5
Family	≥ 86.5
Order	≥ 82.0
Class	≥ 78.5
Phylum	≥ 75.0

Table S4. Library concentrations and input volumes for metatranscriptomic sequencing

Sample ID	concentrations (ng/ul)	volumes(ul)
$+^{15}NO_3^-$-1	8.0	30
$+^{15}NO_3^-$-2	8.5	30
$+^{15}NO_3^-$-3	4.1	30
$+^{15}N_2O$-1	5.1	30
$+^{15}N_2O$-2	6.2	30
$+^{15}N_2O$-3	5.1	30

Table S5. Quality assessment of raw metatranscriptomic reads

Sample ID	Paired read numbers	Total bp numbers	>=Q20(%)	>=Q30(%)
$+^{15}NO_3^-$-1	24,054,792	9,621,916,800	95.8	89.6
$+^{15}NO_3^-$-2	27,355,346	10,942,138,400	95.9	89.7
$+^{15}NO_3^-$-3	25,322,046	10,128,818,400	95.2	88.3
$+^{15}N_2O$-1	26,543,074	7,962,922,200	98.1	94.3
$+^{15}N_2O$-2	26,716,066	8,014,819,800	98.0	94.0
$+^{15}N_2O$-3	24,419,906	7,325,971,800	98.0	93.8

Table S6. Key nitrogen transformation pathways and the corresponding functional genes

Nitrogen Process	Functional genes	Enzymes/Proteins
NO_3^- assimilation	***NRT2***	MFS transporter, NNP family, nitrate/nitrite transporter
	nrtA	nitrate/nitrite transport system substrate-binding protein
	nrtB	nitrate/nitrite transport system permease protein
	nrtC/nrtD	nitrate/nitrite transport system ATP-binding protein
NO_2^- assimilation	***NIT-6***	nitrite reductase (NAD(P)H)
	nirA	ferredoxin-nitrite reductase
	nasB/nasD	nitrite reductase [NAD(P)H] large subunit
	nasE	nitrite reductase [NAD(P)H] small subunit
$NO_3^- \rightarrow NO_2^-$	***narG***	nitrate reductase / nitrite oxidoreductase, alpha subunit
	narH	nitrate reductase / nitrite oxidoreductase, beta subunit
	narI	nitrate reductase gamma subunit
	napA	nitrate reductase (cytochrome)
	napB	nitrate reductase (cytochrome), electron transfer subunit
$NO_2^- \rightarrow NH_4^+$	***nrfA***	nitrite reductase (cytochrome c-552)
	nrfH	cytochrome c nitrite reductase small subunit
	nirB	nitrite reductase (NADH) large subunit
	nirD	nitrite reductase (NADH) small subunit
NH_4^+ transporter	***amt***	ammonium transporter, Amt family
$NO_2^- \rightarrow NO$	***nirK***	nitrite reductase (NO-forming)
	nirS	nitrite reductase (NO-forming) / hydroxylamine reductase
$NO \rightarrow N_2O$	***norB***	nitric oxide reductase subunit B
	norC	nitric oxide reductase subunit C
$N_2O \rightarrow N_2$	***nosZ***	nitrous-oxide reductase
$N_2 \rightarrow NH_4^+$	***nifD***	nitrogenase molybdenum-iron protein alpha chain
	nifK	nitrogenase molybdenum-iron protein beta chain
	nifH	nitrogenase iron protein NifH
	anfG	nitrogenase delta subunit
	vnfD	vanadium-dependent nitrogenase alpha chain
	vnfK	vanadium-dependent nitrogenase beta chain

	vnfG	vanadium nitrogenase delta subunit
	vnfH	vanadium nitrogenase iron protein
$NH_4^+ \rightarrow NO_2^-$ / NO_3^-<br>$NH_4^+ \rightarrow NH_2OH$	***amoA***	methane/ammonia monooxygenase subunit A
	amoB	methane/ammonia monooxygenase subunit B
	amoC	methane/ammonia monooxygenase subunit C
$NH_2OH \rightarrow NO_2^-$	***hao***	hydroxylamine dehydrogenase
